# Phylogenetic and antigenic characterization of reassortant H9N2 avian influenza viruses isolated from wild waterfowl in the East Dongting Lake wetland in 2011–2012

**DOI:** 10.1186/1743-422X-11-77

**Published:** 2014-04-30

**Authors:** Yun Zhu, Shixiong Hu, Tian Bai, Lei Yang, Xiang Zhao, Wenfei Zhu, Yiwei Huang, Zhihong Deng, Hong Zhang, Zhiyong Bai, Mingdong Yu, Jianfei Huang, Yuelong Shu

**Affiliations:** 1 Key Laboratory for Medical Virology, National Health and Family Planning Commission, National Institute for Viral Disease Control and Prevention, China CDC, Beijing 102206, China; 2 Hunan Provincial Center for Disease Control and Prevention, Changsha, China; 3 Yueyang Center for Disease Control and Prevention, Yueyang, China; 4 Hunan East Dongting Lake National Nature Reserve, Yueyang, China; 5 Yueyang County Center for Disease Control and Prevention, Yueyang, China

**Keywords:** Avian influenza virus, Wild waterfowl, H9N2 subtype, Dongting Lake, Reassortant

## Abstract

**Background:**

Wild waterfowl are recognized as the natural reservoir for influenza A viruses. Two distinct lineages, the American and Eurasian lineages, have been identified in wild birds. Gene flow between the two lineages is limited. The H9N2 virus has become prevalent in poultry throughout Eurasia, and mainly circulates in wild ducks and shorebirds in North America.

**Methods:**

In this study, 22 H9N2 avian influenza viruses were isolated from wild waterfowl feces in East Dongting Lake Nature Reserve in November 2011 and March 2012. The phylogenetic, molecular, and antigenic characteristics of these viruses were analyzed based on analyses of the whole genome sequence of each isolate.

**Results:**

Phylogenetic analyses indicated that these H9N2 viruses were generated by reassortment events. The HA, NA, PA, and NS genes were derived from the American gene pool, and the other four genes were derived from the Eurasian gene pool. Antigenic analyses indicated that these viruses were significantly different from the Eurasian lineage viruses.

**Conclusions:**

This study presents the isolation of novel intercontinental recombinant H9N2 viruses from wild waterfowl in the East Dongting Lake wetland. The novel genotype H9N2 virus has not been detected in poultry in the region yet, and may be transmitted to naïve birds in poultry farms. Therefore, our results highlight the need for ongoing surveillance of wild birds and poultry in this region.

## Background

Influenza A viruses can be divided into different subtypes based upon the two surface glycoproteins, hemagglutinin (HA) and neuraminidase (NA) [1,2]. Wild waterfowl are recognized as the natural reservoir of influenza A viruses, especially low pathogenic avian influenza virus (LPAIV) [3]. To date, all of the HA (H1–H16) and NA (N1–N9) subtype avian influenza viruses (AIVs) have been identified in wild waterfowl, with the exceptions of H17N10 and H18N11, which were isolated from bats [2,4,5]. Previous studies indicated that migratory birds play an important role in the emergence of epidemics in birds, pigs, horses, and humans [1]. Generally, AIVs are nonpathogenic in wild birds, although they sometimes cause significant morbidity and mortality when transmitted to domestic poultry [6-8].

The H9N2 subtype influenza virus was first isolated from a turkey in Wisconsin in 1966. It has been most prevalent in wild ducks and shorebirds and has shown no evidence of establishing a stable lineage in land-based poultry in North America [9]. Since the l990s, H9N2 subtype influenza viruses have become prevalent in land-based poultry across East Asia, Middle Asia, Europe and Africa. Epidemiological and phylogenetic studies indicate that three distinct sublineages of H9N2 subtype influenza viruses have been established: Ck/Bj/94-like (A/chicken/Beijing/1/94 or A/duck/Hongkong/Y280/1997), G1-like (A/Quail/Hong Kong/G1/1997) and Korea-like (A/chicken/Y439/1997 or A/chicken/Korea/383490-p96323/1996) [10-12].

Poultry infected with H9N2 subtype AIVs have reduced egg production and moderate morbidity when co-infected with other viruses or bacteria [13]. Since 1999, some H9N2 viruses have been identified with potential human virus receptor specificity, and have been occasionally transmitted to human and swine [14-16]. Moreover, H9N2 viruses may have contributed internal genes to the highly pathogenic avian influenza (HPAI) H5N1 virus in Hong Kong in 1997 and the novel H7N9 avian influenza virus in mainland China in 2013 [17,18]. Therefore, the H9N2 subtype avian influenza viruses have been classified as candidate viruses with pandemic potential [19,20]. The threat to the poultry industry and public health posed by H9N2 subtype avian influenza viruses should not be ignored.

Hunan East Dongting Lake Nature Reserve is one of the largest wetlands in mainland China and is an important overwintering area and staging site for migratory birds that fly along the East Asia–Australia flyway [21]. Moreover, numerous duck farms are found around the lake [22,23]. Every migrating season, wild birds congregate at the lake where they share a common habitat with domestic ducks, which provides an opportunity for virus reassortment. During active surveillance of AIVs between 2011 and 2012, we isolated H9N2 viruses from wild waterfowl in East Dongting Lake wetlands in November 2011 (7 isolates) and March 2012 (15 isolates). The whole genome sequences of all 22 isolates were obtained, and phylogenetic trees for each gene segment were generated to analyze the relationship of these isolates with other circulating viruses in wild birds or poultry. Furthermore, we performed antigenic analyses to investigate the antigenic characteristics of the isolates.

## Results

### Virus isolation and sequence analysis

In total, 6621 environmental samples were collected in Hunan East Dongting Lake wetland. H9N2 subtype avian influenza viruses were isolated in November 2011 (7 isolates) and March 2012 (15 isolates) from wild waterfowl feces (Table 1). The whole genome sequence of each isolate was obtained. The complete viral genome consists of 8 gene segments of negative-sense, single-stranded RNA, including PB2 (2341 bp), PB1 (2341 bp), PA (2233 bp), HA (1742 bp), NP (1565 bp), NA (1467 bp), M (1027 bp), and NS (890 bp).

**Table 1:** The 22 H9N2 AIVs isolated from wild waterfowl feces in the Hunan East Dongting Lake Nature Reserve in 2011–2012

**NO**	**Virus**	**Abbreviation**	**Host**	**Date of Collection**	**GenBank accession numbers**
1	A/wild waterfowl/Dongting/C2032 2011 (H9N2)	Wwf/DT/C2032/2011	wild waterfowl feces	November 2011	KF971946 - KF971953
2	A/wild waterfowl/Dongting/C2123 2011 (H9N2)	Wwf/DT/C2123/2011	wild waterfowl feces	November 2011	KF971954 - KF971961
3	A/wild waterfowl/Dongting/C2148 2011 (H9N2)	Wwf/DT/C2148/2011	wild waterfowl feces	November 2011	KF971962 - KF971969
4	A/wild waterfowl/Dongting/C2149 2011 (H9N2)	Wwf/DT/C2149/2011	wild waterfowl feces	November 2011	KF971970 - KF971977
5	A/wild waterfowl/Dongting/C2150 2011 (H9N2)	Wwf/DT/C2150/2011	wild waterfowl feces	November 2011	KF971978 - KF971985
6	A/wild waterfowl/Dongting/C2203 2011 (H9N2)	Wwf/DT/C2203/2011	wild waterfowl feces	November 2011	KF971986 - KF971993
7	A/wild waterfowl/Dongting/C3109 2011 (H9N2)	Wwf/DT/C3109/2011	wild waterfowl feces	November 2011	KF971994 - KF972001
8	A/wild waterfowl/Dongting/C4296 2012 (H9N2)	Wwf/DT/C4296/2012	wild waterfowl feces	March 2012	KF972002 - KF972009
9	A/wild waterfowl/Dongting/C4316 2012 (H9N2)	Wwf/DT/C4316/2012	wild waterfowl feces	March 2012	KF972010 - KF972017
10	A/wild waterfowl/Dongting/C4317 2012 (H9N2)	Wwf/DT/C4317/2012	wild waterfowl feces	March 2012	KF972018 - KF972025
11	A/wild waterfowl/Dongting/C4326 2012 (H9N2)	Wwf/DT/C4326/2012	wild waterfowl feces	March 2012	KF972026 - KF972033
12	A/wild waterfowl/Dongting/C4327 2012 (H9N2)	Wwf/DT/C4327/2012	wild waterfowl feces	March 2012	KF972034 - KF972041
13	A/wild waterfowl/Dongting/C4329 2012 (H9N2)	Wwf/DT/C4329/2012	wild waterfowl feces	March 2012	KF972042 - KF972049
14	A/wild waterfowl/Dongting/C4429 2012 (H9N2)	Wwf/DT/C4429/2012	wild waterfowl feces	March 2012	KF972050 - KF972057
15	A/wild waterfowl/Dongting/C4430 2012 (H9N2)	Wwf/DT/C4430/2012	wild waterfowl feces	March 2012	KF972058 - KF972065
16	A/wild waterfowl/Dongting/PC2539 2012 (H9N2)	Wwf/DT/PC2539/2012	wild waterfowl feces	March 2012	KF972066 - KF972073
17	A/wild waterfowl/Dongting/PC2540 2012 (H9N2)	Wwf/DT/PC2540/2012	wild waterfowl feces	March 2012	KF972074 - KF972081
18	A/wild waterfowl/Dongting/PC2553 2012 (H9N2)	Wwf/DT/PC2553/2012	wild waterfowl feces	March 2012	KF972082 - KF972089
19	A/wild waterfowl/Dongting/PC2559 2012 (H9N2)	Wwf/DT/PC2559/2012	wild waterfowl feces	March 2012	KF972090 - KF972097
20	A/wild waterfowl/Dongting/PC2560 2012 (H9N2)	Wwf/DT/PC2560/2012	wild waterfowl feces	March 2012	KF972098 - KF972105
21	A/wild waterfowl/Dongting/PC2562 2012 (H9N2)	Wwf/DT/PC2562/2012	wild waterfowl feces	March 2012	KF972106 - KF972113
22	A/wild waterfowl/Dongting/PC2574 2012 (H9N2)	Wwf/DT/PC2574/2012	wild waterfowl feces	March 2012	KF972114 - KF972121

Complete sequences of the 22 H9N2 viruses showed that they were over 99% nucleotide identity in all eight gene segments. Therefore, we selected a representative virus, A/Wild waterfowl/Dongting/C2148/2011 (H9N2) (C2148), for further analysis. Each of the 8 gene segments of C2148 had the highest nucleotide (over 99%) identity with those of A/Egret/Hunan/1/2012 (H9N2), which was isolated in the same region [24]. Additionally, the following genes showed 99% homology with the indicated reference strain: the PB2 gene of C2148 with A/wild duck/Korea/SNU50-5/2009 (H5N1), the PB1 gene of C2148 with A/wild duck/Korea/CSM4-28/2010 (H4N6), the PA gene with A/northern shoveler/California/2810/2011 (H11N2), the HA gene with A/northern shoveler/Interior Alaska/8BM3470/2008 (H9N2), the NP gene with A/duck/Nanjing/1102/2010 (H4N8), the NA gene with A/snow goose/Montana/466771-4/2006 (H5N2), and the M gene with A/wild duck/Korea/CSM4-28/2010 (H4N6). The NS gene showed 98% nucleotide similarity with that of A/surface water/Minnesota/W07-2241/2007 (H3N8) (Table 2).

**Table 2:** Nucleotide Identity (%) of A/Wild waterfowl/Dongting/C2148/2011 H9N2 virus with the closely related isolates in GenBank Database

**Segement**	**Viruses in the GenBank showing high similarity**	**Nucleotide identity (%)**	**Accession Number**
**PB2**	A/wild duck/Korea/SNU50-5/2009 (H5N1)	99%	JX497765
**PB1**	A/wild duck/Korea/CSM4-28/2010 (H4N6)	99%	JX454696
**PA**	A/northern shoveler/California/2810/2011 (H11N2)	99%	CY133906
**HA**	A/northern shoveler/Interior Alaska/8BM3470/2008 (H9N2)	99%	CY079646
**NP**	A/duck/Nanjing/1102/2010 (H4N8)	99%	KC683704
**NA**	A/snow goose/Montana/466771-4/2006 (H5N2)	99%	GQ923263
**M**	A/wild duck/Korea/CSM4-28/2010 (H4N6)	99%	JX454691
**NS**	A/surface water/Minnesota/W07-2241/2007 (H3N8)	98%	CY073724

Comparisons were performed by using the BLAST search in the Influenza Sequcence Database.

### Phylogenetic analyses

**Figure 1:**
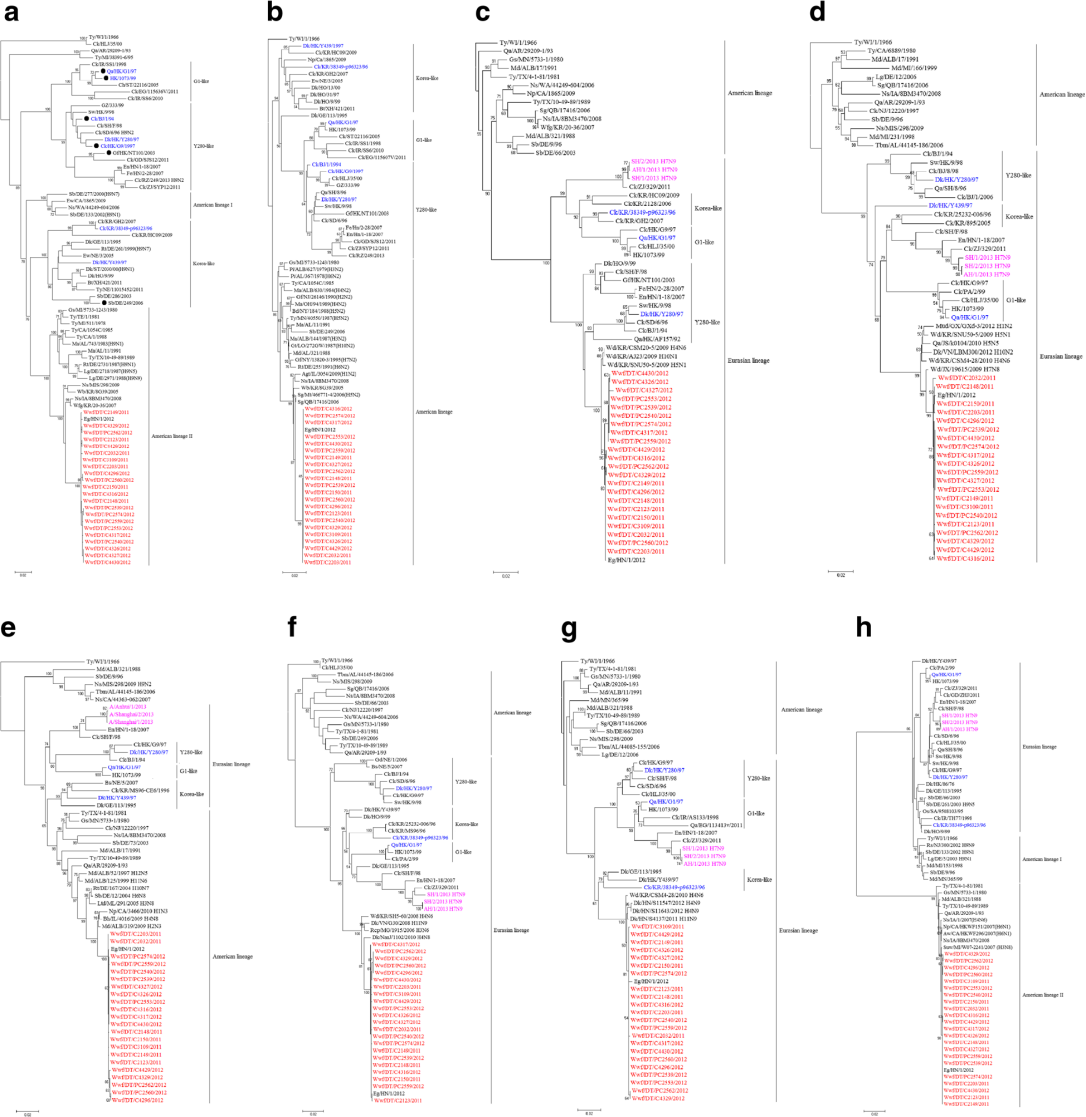
**Phylogenetic trees of HA (a), NA (b), PB2 (c), PB1 (d), PA (e), NP (f), M (g), and NS (h) genes of H9N2 subtype AIVs.** Neighbor-joining (NJ) trees were generated using MEGA 5.01. Estimates of the phylogenies were calculated by performing 1000 neighbor-joining bootstrap replicates, all rooted to the sequence of Turkey/Wisconsin/1/66 (H9N2). Our 22 isolates are highlighted in red and representative strains are shown in blue. Abbreviations: Agt, American green-winged teal; Aw, American wigeon; Bd, black duck; Bh, bufflehead; Bs, Bewick’s swan; Bt, baikal teal; Ch, chukar; Ck, chicken; Dk, duck; Eg, egret; En, environment; Ew, Eurasian wigeon; Fe, feces; Gw, gadwall; Gf, guinea fowl; Gs, goose; Gt, green-winged teal; Lg, laughing gull; Ltd, longtail duck; Md, mallard; Mud, Muscovy duck; Np, northern pintail; Ns, northern shoveler; Os, Ostrich; Pfg, pink-footed goose; Pi, pintail duck; Qa, quail; Rcp, red crested pochard; Rt, ruddy turnstone; Sb, Shorebird; Sg, snow goose; Sw, swine; Suw, surface water; Tbm, thick-billed murre; Ty, turkey; Wb, wild bird; Wd, wild duck; Wfg, white-fronted goose; AH, Anhui; AL, Alaska; ALB, Alberta; AR, Arkansas; BJ, Beijing; CA, California; DE, Delaware; EG, Egypt; GD, Guangdong; GE, Germany; GX, Guangxi; GZ, Guangzhou; HLJ, Heilongjiang; HK, Hong Kong; HN, Hunan; HO, Hokkaido; IA, Interior Alaska; IL, Illinois; IR, Iran; JX, Jiangxi; Js, Jiangsu; KR, Korea; LO, Louisiana; MG, Mongolia; MI, Minnesota; MIS, Missouri; MT, Montana; ML, Maryland; NanJ, Nanjing; NE, Netherlands; NJ, New Jersey; OH, Ohio; PA, Pakistan; QB, Quebec; RZ, Rizhao; SA, South Africa; SD, Shandong; SH, Shanghai; ST, Shantou; TX, Texas; VN, Vietnam; WA, Washington; Wi, Wisconsin; XH, Xianghai; ZJ, Zhejiang.

To further characterize the evolution of the 22 H9N2 viruses isolated from wild waterfowl, phylogenetic trees for each gene were constructed. In the HA gene tree, the H9 subtype AIVs clustered into five distinct lineages: G1-like, Y280-like, Korea-like, and two American lineages. The strains that clustered with the G1-like, Y280-like, and Korea-like sublineages are known to have been endemic in domestic poultry in Eurasia and Africa [25]. All of the 22 isolates belonged to the American lineage II, which included viruses that are prevalent in wild birds or turkeys in North America. Some genetic exchange was observed between the North American and Eurasian strains, as a strain isolated in Korea clustered with an American lineage, and several H9 subtype AIVs isolated in America clustered with the Korea-like sublineage (Figure 1a). Similar evolutionary patterns were observed in the N2 gene tree, which also clustered into four lineages: G1-like, Y280-like, Korea-like and American lineages. The NA genes of all isolates clustered with the American lineage, and were most closely related to an H5N2 AIV subtype isolated from a snow goose in Montana (Figure 1b). The PA and NS genes of the isolates both clustered with the American lineage and showed high nucleotide similarity with strains isolated from wild birds in North America (Figure 1e and h). By contrast, the PB2, PB1, NP, and M internal genes clustered with the Eurasian gene pool, but were distinct from the H9N2 viruses that circulated in Eurasia and Africa and the corresponding internal genes of the novel H7N9 AIV (Figure 1c, d, f, and g).

**Figure 2:**
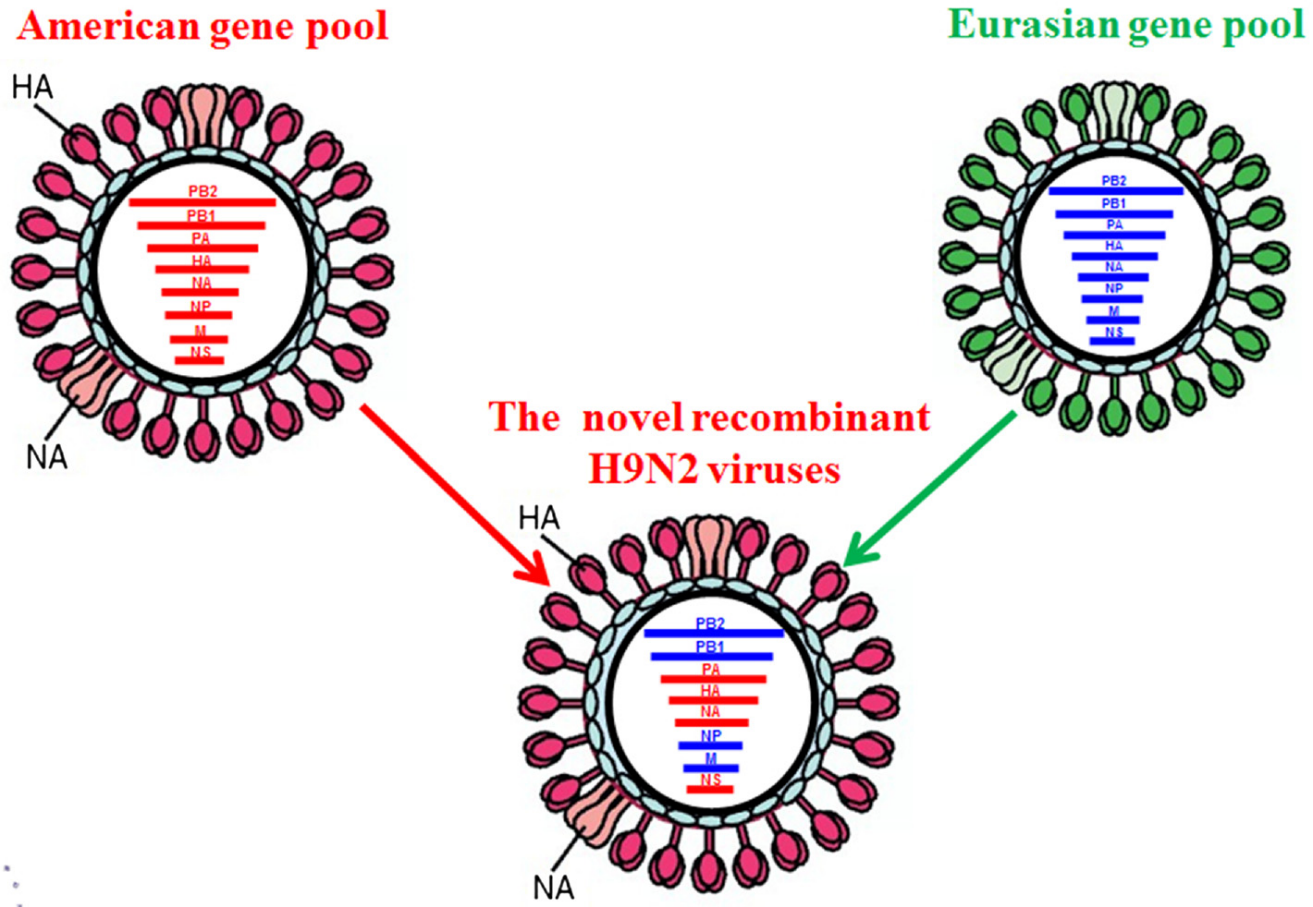
**A hypothetical reassortment pattern of the novel H9N2 virus isolates.** Red colored gene segments indicate genes that are derived from the American AIV gene pool and blue colored gene segments indicate genes that originated in the Eurasian gene pool.

Phylogenetic analyses indicated that these isolates were novel recombinant H9N2 viruses. In these viruses, 4 genes (HA, NA, PA, and NS) were derived from the American AIV gene pool and 4 genes (PB2, PB1, NP, and M) were derived from the Eurasian gene pool (Figure 2).

### Molecular analysis

The molecular characteristics of the 22 H9N2 isolates were compared with representative H9N2 virus strains circulating in Eurasia and America. The amino acid sequence of the cleavage sites of all isolates possessed a single basic amino acid (R) within the HA connecting peptide (VPELPKGR↓GLF), which is a typical feature of LPAIVs [1]. The HA receptor-binding pocket included the avian-like motif, Q226 and G228 (H3 numbering, Table 3), suggesting that these viruses could preferentially bind to an avian-like receptor (α2, 3-linked sialic acid) [26]. Analysis of potential HA protein N-X-S/T glycosylation site motifs revealed 8 sites at positions 29, 82, 141, 218, 298, 305, 492, and 551 (Table 4).

**Table 3:** Amino acid sequences of specific sites in the HA, NA, and PB2 proteins of 22 H9N2 AIVs

**Virus**	**RBS**^**a**^					**HA cleavage site**	**NA deletion**			**PB2**	
	**183**	**190**	**226**	**227**	**228**		**38-39**	**46-50**	**63-65**	**627**	**701**
A/Turkey/Wisconsin/1/66	H	E	Q	Q	G	PAVSSR↓GLF	NO	NO	NO	E	D
A/chicken/Beijing/1/94	N	V	Q	Q	G	PARSSR↓GLF	NO	NO	NO	E	D
A/quail/Hong Kong/G1/97	H	E	L	Q	G	PARSSR↓GLF	YES	NO	NO	E	D
A/chicken/Hong Kong/G9/97	N	A	L	Q	G	PARSSR↓GLF	NO	NO	NO	E	D
A/duck/Hong Kong/Y280/97	N	T	L	Q	G	PARSSR↓GLF	NO	NO	NO	E	D
A/duck/Hong Kong/Y439/97	H	E	Q	Q	G	PAASNR↓GLF	NO	NO	NO	E	D
A/Chicken/Korea/38349-p96323/96 H9N2	H	E	Q	Q	G	PAASYR↓GLF	NO	NO	NO	E	D
A/Hong Kong/1073/99	H	E	L	Q	G	PARSSR↓GLF	YES	NO	NO	E	D
A/guinefowl/HongKong/NT101/2003	N	A	L	Q	G	PARSSR↓GLF	NO	NO	YES	E	D
A/shorebird/Delaware/249/2006	H	E	Q	Q	G	PAASDR↓GLF	NO	NO	NO	E	D
A/northern shoveler/Interior Alaska/8BM3470/2008	H	E	Q	Q	G	PAASDR↓GLF	NO	NO	NO	E	D
A/egret/Hunan/1/2012	H	E	Q	Q	G	PAASDR↓GLF	NO	NO	NO	E	D
A/wild waterfowl/Dongting/C2032/2011	H	E	Q	Q	G	PAASDR↓GLF	NO	NO	NO	E	D
A/wild waterfowl/Dongting/C2123/2011	H	E	Q	Q	G	PAASDR↓GLF	NO	NO	NO	E	D
A/wild waterfowl/Dongting/C2148/2011	H	E	Q	Q	G	PAASDR↓GLF	NO	NO	NO	E	D
A/wild waterfowl/Dongting/C2149/2011	H	E	Q	Q	G	PAASDR↓GLF	NO	NO	NO	E	D
A/wild waterfowl/Dongting/C2150/2011	H	E	Q	Q	G	PAASDR↓GLF	NO	NO	NO	E	D
A/wild waterfowl/Dongting/C2203/2011	H	E	Q	Q	G	PAASDR↓GLF	NO	NO	NO	E	D
A/wild waterfowl/Dongting/C3109/2011	H	E	Q	Q	G	PAASDR↓GLF	NO	NO	NO	E	D
A/wild waterfowl/Dongting/C4296/2012	H	E	Q	Q	G	PAASDR↓GLF	NO	NO	NO	E	D
A/wild waterfowl/Dongting/C4316/2012	H	E	Q	Q	G	PAASDR↓GLF	NO	NO	NO	E	D
A/wild waterfowl/Dongting/C4317/2012	H	E	Q	Q	G	PAASDR↓GLF	NO	NO	NO	E	D
A/wild waterfowl/Dongting/C4326/2012	H	E	Q	Q	G	PAASDR↓GLF	NO	NO	NO	E	D
A/wild waterfowl/Dongting/C4327/2012	H	E	Q	Q	G	PAASDR↓GLF	NO	NO	NO	E	D
A/wild waterfowl/Dongting/C4329/2012	H	E	Q	Q	G	PAASDR↓GLF	NO	NO	NO	E	D
A/wild waterfowl/Dongting/C4429/2012	H	E	Q	Q	G	PAASDR↓GLF	NO	NO	NO	E	D
A/wild waterfowl/Dongting/C4430/2012	H	E	Q	Q	G	PAASDR↓GLF	NO	NO	NO	E	D
A/wild waterfowl/Dongting/PC2539/2012	H	E	Q	Q	G	PAASDR↓GLF	NO	NO	NO	E	D
A/wild waterfowl/Dongting/PC2540/2012	H	E	Q	Q	G	PAASDR↓GLF	NO	NO	NO	E	D
A/wild waterfowl/Dongting/PC2553/2012	H	E	Q	Q	G	PAASDR↓GLF	NO	NO	NO	E	D
A/wild waterfowl/Dongting/PC2559/2012	H	E	Q	Q	G	PAASDR↓GLF	NO	NO	NO	E	D
A/wild waterfowl/Dongting/PC2560/2012	H	E	Q	Q	G	PAASDR↓GLF	NO	NO	NO	E	D
A/wild waterfowl/Dongting/PC2562/2012	H	E	Q	Q	G	PAASDR↓GLF	NO	NO	NO	E	D
A/wild waterfowl/Dongting/PC2574/2012	H	E	Q	Q	G	PAASDR↓GLF	NO	NO	NO	E	D

^a^According to the H3 numbering.

**Table 4:** The comparison of HA amino acid sequences of H9N2 AIVs

**Virus**	**Potential glycosylation sites**									
	**29-31**	**82-84**	**105-107**	**141-143**	**206-208**	**218-220**	**298-300**	**305-307**	**492-494**	**551-553**
A/Turkey/Wisconsin/1/66	NST	NPS	-	NVT	-	NRT	NTT	NIS	NGT	NGS
A/chicken/Beijing/1/94	NST	NPS	-	NVT	-	NRT	NTT	NVS	NGT	NGS
A/quail/Hong Kong/G1/97	NST	NPS	NGT	NVT	NDT	NRT	NST	NIS	NGT	-
A/chicken/Hong Kong/G9/97	NST	NPS	-	NVS	-	NRT	NTT	NVS	NGT	NGS
A/duck/Hong Kong/Y280/97	NST	NPS	-	NVS	-	NRT	NTT	NVS	NGT	-
A/duck/Hong Kong/Y439/97	NST	NPS	-	NVT	-	NRT	NTT	NVS	NGT	NGS
A/Hong Kong/1073/99	NST	NPS	NGT	NVT	NDT	NRT	NST	NIS	NGT	NGS
A/guinefowl/HongKong/NT101 2003	NST	NPS	-	NVS	-	NRT	NTT	NVS	NGT	NGS
A/shorebird/Delaware/249/2006	NST	-	NGT	NVT	-	NRT	NTT	NVS	NGT	NGS
A/wild waterfowl/Dongting/C2032/2011	NST	NPS	-	NVT	-	NRT	NTT	NVS	NGT	NGS
A/wild waterfowl/Dongting/C2123/2011	NST	NPS	-	NVT	-	NRT	NTT	NVS	NGT	NGS
A/wild waterfowl/Dongting/C2148/2011	NST	NPS	-	NVT	-	NRT	NTT	NVS	NGT	NGS
A/wild waterfowl/Dongting/C2149/2011	NST	NPS	-	NVT	-	NRT	NTT	NVS	NGT	NGS
A/wild waterfowl/Dongting/C2150/2011	NST	NPS	-	NVT	-	NRT	NTT	NVS	NGT	NGS
A/wild waterfowl/Dongting/C2203/2011	NST	NPS	-	NVT	-	NRT	NTT	NVS	NGT	NGS
A/wild waterfowl/Dongting/C3109/2011	NST	NPS	-	NVT	-	NRT	NTT	NVS	NGT	NGS
A/wild waterfowl/Dongting/C4296/2012	NST	NPS	-	NVT	-	NRT	NTT	NVS	NGT	NGS
A/wild waterfowl/Dongting/C4316/2012	NST	NPS	-	NVT	-	NRT	NTT	NVS	NGT	NGS
A/wild waterfowl/Dongting/C4317/2012	NST	NPS	-	NVT	-	NRT	NTT	NVS	NGT	NGS
A/wild waterfowl/Dongting/C4326/2012	NST	NPS	-	NVT	-	NRT	NTT	NVS	NGT	NGS
A/wild waterfowl/Dongting/C4327/2012	NST	NPS	-	NVT	-	NRT	NTT	NVS	NGT	NGS
A/wild waterfowl/Dongting/C4329/2012	NST	NPS	-	NVT	-	NRT	NTT	NVS	NGT	NGS
A/wild waterfowl/Dongting/C4429/2012	NST	NPS	-	NVT	-	NRT	NTT	NVS	NGT	NGS
A/wild waterfowl/Dongting/C4430/2012	NST	NPS	-	NVT	-	NRT	NTT	NVS	NGT	NGS
A/wild waterfowl/Dongting/PC2539/2012	NST	NPS	-	NVT	-	NRT	NTT	NVS	NGT	NGS
A/wild waterfowl/Dongting/PC2540/2012	NST	NPS	-	NVT	-	NRT	NTT	NVS	NGT	NGS
A/wild waterfowl/Dongting/PC2553/2012	NST	NPS	-	NVT	-	NRT	NTT	NVS	NGT	NGS
A/wild waterfowl/Dongting/PC2559/2012	NST	NPS	-	NVT	-	NRT	NTT	NVS	NGT	NGS
A/wild waterfowl/Dongting/PC2560/2012	NST	NPS	-	NVT	-	NRT	NTT	NVS	NGT	NGS
A/wild waterfowl/Dongting/PC2562/2012	NST	NPS	-	NVT	-	NRT	NTT	NVS	NGT	NGS
A/wild waterfowl/Dongting/PC2574/2012	NST	NPS	-	NVT	-	NRT	NTT	NVS	NGT	NGS

There were no deletions in the NA stalk region, and no H274Y or N294S substitutions were observed in the NA protein. These properties indicated that these viruses would be sensitive to NA inhibitors, such as oseltamivir [27,28]. They encoded amino acids E and D at positions 627 and 701 of the PB2 protein, respectively, which are characteristics of AIVs [29-31]. No amino acid mutations associated with amantadine resistance were observed in the M2 ion channel protein. Additionally, no substitutions associated with increased virulence in mammals were detected in the PB2, PB1, or NS proteins (data not shown).

### Antigenic analysis

To assess the antigenic properties of our novel H9N2 isolates, we performed Hemagglutination inhibition (HI) assays with 5 antisera raised against representative H9N2 viruses and two antisera raised against H9N2 isolates from this study (C2148 and PC2539). The HI antibody titers that recognized the C2148 and PC2539 viruses were much higher than the titers against the other H9N2 representative viruses, including the antiserum that was raised against a virus isolated from shorebirds in America (Sb/DE/249/06) (Table 5). These results indicated that the H9N2 viruses isolated in this study were antigenically distinct from the previously identified H9N2 viruses.

**Table 5:** Cross-reactivity (HI-determined titers) of the H9N2 AIVs with sera against represented strains

**Virus**	**Ferret antisera against H9N2 AIV**							
	**Ck/BJ**	**G9**	**G1**	**HK/1073**	**NT101**	**DE/249**	**C2148**	**PC2539**
Ck/BJ	**640**	160	<10	80	160	<10	320	160
G9	640	**1280**	40	80	1280	160	320	160
G1	<10	<10	**2560**	640	<10	<10	<10	<10
HK/1073	40	<10	1280	**1280**	<10	20	160	40
NT101	640	2560	160	160	**1280**	320	640	160
DE/249	<10	<10	<10	<10	<10	**80**	<10	<10
C2148	<10	<10	<10	<10	<10	<10	**1280**	160
PC2539	<10	<10	<10	<10	<10	<10	1280	**320**

Homologous titers are bold.

## Discussion

It is generally accepted that wild waterfowl are the natural reservoir for AIVs, and play important roles in the perpetuation and dissemination of AIVs, especially LPAIV. Previous surveillance studies indicated that AIVs circulate in diverse bird species, including *Anseriforms* (ducks, geese, and swans) and *Charadriiforms* (gulls and terns). AIVs preferentially infect cells that line the intestinal tract and numerous viruses can be excreted at high concentrations in wild waterfowl feces. Influenza viruses remain infectious in lake water for up to 4 days at 22°C and for over 30 days at 0°C. Contaminated lake water may result in efficient transmission to naïve birds by the fecal-oral route [32-34]. Migrating birds annually travel between breeding and overwintering sites, so AIVs harbored by migrating birds can be distributed along the migration flyway. The Hunan East Dongting Lake Nature Reserve is located along the East Asia–Australia flyway and is a major staging and overwintering site for migratory birds. Moreover, many domestic duck farms are located in this region and employ a free-range style to raise domestic ducks [22]. Because wild waterfowl and domestic ducks may share common habitats, water, and food, genetic exchange between different subtypes of AIVs circulating in wild waterfowl and domestic poultry may be possible in this location [35].

In this study, we obtained 22 H9N2 subtype AIV isolates from wild waterfowl in East Dongting Lake wetland in November 2011 and March 2012. Genetic analyses showed that the 22 H9N2 viruses shared over 99% nucleotide sequence homology with the strain A/Egret/Hunan/1/2012 (H9N2) in all eight gene segments, which was isolated from an egret in the same region in January 2012. These findings indicated that these viruses had a common origin. Long-term surveillance studies in America and Eurasia demonstrated that the most prevalent AIVs in wild birds could be separated into Eurasian and American lineages. While intercontinental virus exchange existed in migrating birds, its frequency was limited and invasions by whole viruses were not observed [36]. Instances of invasion by the American lineage H9N2 virus into the Eurasian continent have been reported [24]. Notably, the 22 isolates reported in this study were novel recombinant H9N2 subtype AIVs, which encoded genes derived from both the American and Eurasian gene pools circulating in wild birds. Therefore, genes that originated from the American lineage may have been carried by migratory birds from North America, leading to the generation of multiple AIV reassortants circulating in wild birds in Eurasia. Moreover, this recombinant genotype was detected both by us and Chen and colleagues in different wild birds in three months during 2011–2012 (November 2011, January 2012, and March 2012), suggesting that this virus has been circulating in wild birds in this region [24]. Although H9N2 viruses of this genotype have not been reported in other regions, they may have been distributed to other places by migratory birds along their flyways.

H9N2 AIVs have been endemic in domestic poultry across Eurasia and Africa since the late 1990s. The wide circulation of H9N2 viruses provides more opportunities for reassortment with other AIV subtypes in poultry. In the last few decades, H9N2 viruses may have provided internal genes to the HPAI H5N1 and the novel H7N9 avian influenza viruses [17,18]. Additionally, H9N2 viruses have been occasionally transmitted from poultry to mammals (including humans and swine), and some of the H9N2 viruses showed the ability to bind efficiently to α2, 6-linked sialic acid, which can indicate human virus receptor specificity [15,26]. Further studies indicated that H9N2 viruses that encoded Leu226 could replicate in ferrets and be transmitted by direct contact [37]. Therefore, many researchers believe that the H9N2 viruses have the potential to cause a future pandemic. Even if they do not directly cause a pandemic, they could indirectly contribute to an influenza pandemic by contributing internal genes to a reassortant virus [17,18,38]. Our findings demonstrated that all 22 isolates were LPAIVs, and we observed neither G226L substitutions in HA proteins nor any other pathogenicity-associated mutations in other viral proteins. Nevertheless, the pathogenicity of these viruses should be further accessed in poultry and mammalian animal models.

Antigenic analyses suggested that the antigenicity of the isolates were significantly different from the G1- and Y280-like viruses, which have been dominant in domestic poultry in Eurasia and Africa, and the “America” virus, which is closely related to the Korea-like viruses. The antigenic and phylogenetic analyses were generally consistent with each other. Importantly, poultry in mainland China have been widely vaccinated with commercial inactivated vaccines that contain representative prevalent influenza strains circulating in poultry in Eurasia, but not strains of the American lineage. Therefore, the currently used commercial vaccines may not protect poultry or prevent the transmission of the novel H9N2 subtype viruses in China.

## Conclusions

In summary, the 22 H9N2 viruses isolated from wild waterfowl in 2011–2012 were novel reassorted H9N2 subtype AIVs with similar genotypes. All isolates encoded genes for proteins with low pathogenic characteristics. Determining whether these H9N2 AIVs can transmit to poultry or other animals or further adapt to new hosts will require continuous monitoring in the future. Our findings extend our knowledge of the ecology of AIVs circulating in wild birds in the Dongting Lake region and highlight the importance of intercontinental AIV gene flow in migratory birds. Therefore, we emphasize the vital need for continued surveillance of AIVs in wild birds and poultry to prepare for and respond to potential influenza pandemics.

## Materials and methods

### Sample collection

During November 2011 and April 2012, we collected fresh fecal samples from wild birds and lake water in Hunan East Dongting Lake Nature Reserve (28°58′–29°38′N, 112°43′–113°13′E), Yueyang, Hunan, China. This site is an important overwintering habitat for East Asian migratory birds that is located in the middle reaches of the Yangtze River. Fresh fecal samples were collected with sterile cotton swabs, following previously described protocols [39], and placed in 15 mL tubes containing 4 mL virus transport media (VTM). The VTM contained: Tissue culture medium 199 (Thermo Scientific Hyclone, Logan, UT, USA), 0.5% BSA (Roche, Mannheim, Germany), 10% glycerol, 2 × 10^6^ U/L penicillin G, 200 mg/L streptomycin, 2 × 10^6^ U/L polymyxin B sulfate, 250 mg/L gentamicin, 60 mg/L ofloxacin HCI, 0.2 g/L sulfamethoxazole, and 5 × 10^5^U/L nystatin (Sigma, St Louis, MO, USA). Samples were immediately transported to the laboratory at 4°C and stored at -80°C.

### Virus detection and isolation

RNA was extracted from 200 μL fecal suspension using the BioRobot Universal system with the QIAamp One-For-All Nucleic Acid Kit (Qiagen, Hilden, Germany) in accordance with the manufacturer’s instructions. Influenza A virus was screened using qPCR assay that targeted the influenza Matrix gene. Detection was performed using a Stratagene Mx3005P thermocycler with an AgPath RT-PCR Kit (Applied Biosystems, Foster City, CA, USA) using 5 μL eluate in 25 μL total volume. Each run included 2 negative and 2 positive control samples along with 92 samples. The positive samples detected by qPCR were inoculated into the allantoic cavity of 9-day-old specific pathogen free (SPF) embryonated chicken eggs (ECEs). The ECEs were incubated at 37°C for 48 h and then chilled at 4°C for 6–8 h. Allantoic fluids were harvested and hemagglutination assays with 0.5% turkey red blood cells confirmed the presence of viruses.

### Virus subtyping and sequencing

Viral RNA was extracted from infected allantoic fluid using the RNeasy Mini Kit (Qiagen), and reverse transcribed using the Uni12 primer (5′-AGCAAAAGCAGG-3′) with the SuperRT cDNA Kit (CWBIO, Beijing, China). Isolate subtyping was performed by PCR using 16 sets of HA (H1–H16) primers and 9 sets of NA (N1–N9) primers designed by the Chinese National Influenza Center. Complete genome amplification was performed using specific primers (primer sequences available on request) with 2× Es Taq MasterMix Kit (CWBIO). PCR products of the expected sizes were purified using a QIAquick PCR purification kit (Qiagen). Sequencing was performed using the BigDye Terminator v3.1 Cycle Sequencing Kit with an ABI PRISM 3700xl DNA Analyzer (Applied Biosystems), following the manufacturer’s instructions.

### Phylogenetic analyses

We performed multiple sequence alignments using the MAFFT software, version 6. Both sequences of the representative H9N2 subtype influenza A virus strains circulating in America and Eurasia and homologous sequences that shared high nucleotide similarities with our H9N2 isolates were included in the phylogenetic analyses. Preliminary phylogenetic trees were constructed to infer the overall topology, using more than 500 sequences for each gene. In order to more clearly define the phylogenetic relationships of the 22 H9N2 virus isolates, representative sequences of each cluster were selected to generate neighbor-joining (NJ) trees. Phylogenetic estimates were calculated by performing 1000 neighbor-joining bootstrap replicates.

### Antigenic analyses

Antigenic analyses were performed using 6 polyclonal ferret antisera against A/chicken/Beijing/1/1994 (H9N2) (Ck/Bj), A/quail/Hong Kong/G1/1997 (H9N2) (G1), A/chicken/Hong Kong/G9/1997 (H9N2) (G9), A/Hong Kong/1073/1999 (H9N2) (HK/1073), A/chicken/Hong Kong/NT101/2003 (H9N2) (HK/NT101), and A/shorebird/ Delaware /249/2006 (H9N2) (DE/249), which were kindly provided by Dr. Webby. Polyclonal ferret antisera raised against A/wild waterfowl/Dongting/C2148/2011 (H9N2) (C2148) and A/wild waterfowl/Dongting/PC2539/2012 (H9N2) (PC2539), two representative influenza strains, were also used in our study. HI assays were performed as previously described. Briefly, all sera were treated with receptor destroying enzyme II (RDE) (Denka Seiken, Tokyo, Japan) to remove nonspecific inhibitors of hemagglutination by adding 3 volumes of RDE to tubes with 1 volume of serum. Samples were incubated at 37°C for 16–18 h, and then inactivated at 56°C for 30 min. After RDE inactivation, 6 volumes of phosphate buffered saline (PBS; Thermo Scientific Hyclone) were added. The diluted sera were then serially diluted 2-fold with 25 μL PBS, and equal volumes of antigen (8 HA unit/50 μL) were added to each well. The plates were gently mixed and incubated at room temperature for 20–30 min. Viral titers were determined by adding 50 μL 0.5% turkey red blood cells to each well. The limit of detection for HI titers was ≤ 20.

### Nucleotide sequence accession numbers

The nucleotide sequences generated in our study were deposited at GenBank under the accession numbers KF971946 to KF972121.

## Competing interests

The findings and conclusions of this report are those of the authors and do not necessarily represent the views of the funding agency. We declare no conflict of interest.

## Authors’ contributions

YLS and SXH designed the research; YZ performed research and drafted the manuscript; TB, YWH, ZHD, HZ, ZYB, MDY, and JFH collected samples and performed research; LY and XZ analyzed data; and YLS and WFZ helped to draft and revise the manuscript. All authors read and approved the final manuscript.
